# Severe neonatal hyperbilirubinaemia in European and Indian subcontinent descendent newborns: a retrospective cohort study

**DOI:** 10.1007/s00431-024-05892-x

**Published:** 2024-11-29

**Authors:** João Ferreira Simões, Margarida Simão, Paula Rocha, Sara Ferreira, Rosário Perry da Câmara, Diana Amaral, Beatriz Costa, Mário Coelho

**Affiliations:** Área de Pediatria, Unidade de Pediatria Médica, Hospital Dona Estefânia, Unidade Local de Saúde São José, Centro Académico de Lisboa, 5.1, Lisbon, Portugal

**Keywords:** Neonatal hyperbilirubinemia, Disease severity, Risk factors

## Abstract

Neonatal hyperbilirubinaemia is more common in Asian-descendent populations, but differences in disease severity are poorly reported. Our study aimed to compare neonatal hyperbilirubinaemia severity between European and Indian subcontinent descendent newborns. We conducted a single-centre retrospective cohort study including newborns admitted with unconjugated hyperbilirubinaemia (January 2016 to December 2021). Patients were followed during admission, comparing those with European ancestry (control group) and Indian subcontinent ancestry (India, Pakistan, Bangladesh and Nepal) (study group). The primary outcome was severe hyperbilirubinemia (TSB > 25 mg/dL, phototherapy > 6 h or need for exchange transfusion [ET]), and the secondary was TSB levels. Adjusted analysis for potential confounding factors was performed using binary logistic regression models. Of 110 newborns included, 27 (24.5%) had Indian subcontinent ancestry. Occurrence of TSB > 25 mg/dL was significantly higher in the study group (22.2% vs. 4.8%, *p* = 0.006), while no differences were noted in exposure to phototherapy > 6 h and ET therapy. Logistic regression models for confounding factors adjustment showed Indian subcontinent ancestry as an independent risk factor for TSB > 25 mg/dL (OR 7.49, CI 95% [1.23–45.50]). The study group revealed also higher absolute values of TSB both at admission (22.0 mg/dL vs. 19.6 mg/dL, *p* = 0.013) and at discharge (13.6 mg/dL vs. 11.4 mg/dL, *p* = 0.005).

*Conclusion*: Our findings suggest that newborns with Indian subcontinent ancestry might show a higher risk for the development of severe hyperbilirubinemia compared to European ancestry newborns. Implementing earlier treatment thresholds in this subset of patients may help prevent severe hyperbilirubinemia.

**Table:** 

**What is Known:** • *Indian subcontinent descendent populations have high incidence of neonatal hyperbilirubinaemia but data regarding its severity are scarce.*
**What is New:** • *This article shows that, compared to European descendent newborns, Indian subcontinent descendent newborns might be at higher risk for severe hyperbilirubinaemia.*

**What is Known:**

• *Indian subcontinent descendent populations have high incidence of neonatal hyperbilirubinaemia but data regarding its severity are scarce.*

**What is New:**

• *This article shows that, compared to European descendent newborns, Indian subcontinent descendent newborns might be at higher risk for severe hyperbilirubinaemia.*

## Introduction

Neonatal unconjugated hyperbilirubinaemia is one of the most common reasons for hospitalization in the neonatal period, with reported incidences of 60% and 80% in term and preterm newborns, respectively [1, 2].

Bilirubin high levels in newborns may result from increased haemoglobin production (hemolysis, polycythemia, sepsis), defects in conjugation (Crigler-Najjar syndrome, Gilbert syndrome, 5′-diphosphate-glucuronosyltransferase [UGT] polymorphisms), enterohepatic recirculation increase (breastfeeding, constipation) or decreased clearance (galactosaemia, hypothyroidism) [3–5]. Although, in most cases, it is a benign clinical picture, protein-unbound unconjugated bilirubin can cross the blood–brain barrier, where its deposition may lead to acute and/or chronic neurotoxicity. Bilirubin-induced neurologic dysfunction (BIND) spectrum encompasses a multitude of neurodevelopmental disorders, from mild to severe, including auditory deficits, language disorders, attention deficit hyperactivity disorder, specific learning disorders and cognitive delay [6–8]. Long-term neurological dysfunction may occur in the absence of acute encephalopathy, with higher levels of total serum bilirubin (TSB) (> 20–25 mg/dL) and/or prolonged exposure [2, 9]. The American Academy of Pediatrics (AAP) published new guidelines in 2022 for the management of neonatal hyperbilirubinaemia, defining new nomograms for hyperbilirubinaemia management and higher thresholds for phototherapy and exchange-transfusion (ET) therapy [10].

Neonatal unconjugated hyperbilirubinaemia incidence varies geographically, estimating 12% incidence among African-ancestry newborns, 20% in European-ancestry newborns and 49% in Asian-ancestry newborns [11, 12]. Several different genetic backgrounds have been linked to these asymmetric incidences, particularly involving the UGT1A1 gene [1, 13–15]. High-risk populations for hyperbilirubinaemia refer mainly to children from China, Japan and the Philippines, but data regarding newborns from the Indian subcontinent (India, Pakistan, Bangladesh and Nepal) are scarce [11, 16]. Moreover, studies largely address higher incidence, but few describe differences in clinical severity [17].

This study aims to analyse the clinical outcomes of inpatients with neonatal unconjugated hyperbilirubinaemia comparing newborns from European and Indian subcontinent ancestry, including its severity regarding TSB absolute level, prolonged phototherapy and need for ET.

## Materials and methods

### Design, setting and participants

We conducted a retrospective, longitudinal, single-centre cohort study evaluating neonatal hyperbilirubinaemia severity in Lisbon, Portugal. Potentially, eligible patients included newborns admitted due to indirect hyperbilirubinaemia (codified main diagnosis), while all patients with direct hyperbilirubinaemia (direct bilirubin > 1 mg/dL) were excluded. We compared clinical and laboratory data from newborns of Indian subcontinent countries’ ancestry (India, Nepal, Pakistan and Bangladesh) (study group) with those of European ancestry (control group). Ancestry was assumed only by maternal nationality.

Individuals considered were inpatients in the general paediatrics ward in a tertiary paediatric hospital in Lisbon, Portugal. Enrolled patients were selected during a 6-year period, from January 2016 to December 2021. All patients that met inclusion criteria were included, and no sample size calculation was performed. Patients were followed since admission until discharge. Data collection occurred for 6 months, from January 2022 to June 2022.

The study protocol was approved by the institutional review board and the ethics committee prior to data collection (approval number 1069/2021). Informed consent was waived by the ethical review board. We followed Strengthening the Reporting of Observational Studies in Epidemiology (STROBE) guidelines for reporting.

### Outcomes and variables

The primary outcome was a composite of severe hyperbilirubinaemia (defined by TSB > 25 mg/dL or phototherapy > 6 h or ET). Hyperbilirubinemia severity based on the need for ET and TSB > 25 mg/dL were used since the first is the last and most invasive treatment modality, and TSB > 25 mg/dL is near the ET threshold virtually for any chronological and gestational age according to AAP guidelines [10]. We choose phototherapy duration > 6 h cut-off as the patients are reassessed 4–6 h after initiating phototherapy, and the treatment modality is again redecided by that time [12]. As a secondary outcome, differences in total and direct bilirubin values at each moment of evaluation (admission and discharge) were compared between both groups.

Relevant demographic, laboratory and clinical variables were obtained through individual digital file consultation. Demographic variables considered were age at admission and at discharge, sex, maternal age and maternal nationality. In cases where the mother’s nationality was not registered in the clinical record, the patient was excluded, in order to reduce bias induced by intuitive name interpretation. Laboratory variables studied were TSB and direct bilirubin values at admission, peak values recorded during admission and discharge values, all considered continuous variables. Laboratory measurement of TSB was performed by spectrophotometric assay. Data on intervention were also collected, both phototherapy (and its cumulative duration in hours) and/or ET. Clinical variables collected considered as potential confounding factors included gestational age (preterm, term or post-term), birth route, birth weight (and its percentile for gestational age), simultaneous relevant diagnosis (sepsis, Rh and/or AB0 isoimmunization and glucose 6-phosphate dehydrogenase deficiency), number of siblings (0, 1, 2 and 3 or more), feeding regimen (exclusive breastfeeding, exclusive formula or mixed regimen), maternal smoking habits, gestational diabetes and other familiar risk factors for hyperbilirubinaemia (siblings and/or parents with history of neonatal jaundice phototherapy during neonatal period, haemolytic anaemia or Gilbert’s syndrome). If suitable, genetic test results for UGT1A1-related mutations were also reported.

### Statistical analysis

Categorical variables were reported using frequencies and percentages and continuous variables using means and standard deviation (SD) or median and interquartile range (IQR), when skewly distributed. Normality was tested using Kolmogorov–Smirnov and Shapiro–Wilk tests. Comparison across groups was performed using Chi-square tests for categorical data (Fishers’ when applicable) and *T*-tests or Mann–Whitney *U* for continuous data, whether normally or non-normally distributed. Missing data were handled using the pairwise deletion method. Adjusted analysis was conducted using binary multivariate logistic regression, and the independent variables included in the model were previously chosen based on biological and clinical plausibility (sex, prematurity, family history of hyperbilirubinaemia, Indian subcontinent ancestry and sepsis) as well as those with statistical significance in univariate analysis (age at admission and gestational diabetes). Gestational diabetes showed an abnormally high odds ratio (OR) without statistical significance (OR 355038900.6, *p* = 0.999) and was therefore excluded from the model due to the high risk of multicollinearity. Consequently, covariates included in the final model were sex, prematurity, family history of hyperbilirubinaemia, Indian subcontinent ancestry, sepsis and age at admission, and all showed variance inflation factor < 2.5, excluding significant multicollinearity [18, 19]. The Hosmer–Lemeshow test was used to assess the model’s goodness of fit [18]. Independent risk factors for the development of severe hyperbilirubinaemia were reported with OR and 95% confidence intervals (CI). Statistical analysis was made using SPSS 28.0.1.0 for iOS (IBM SPSS Statistics) and STATA 14.1 IC for Windows (StataCorp LLC). We considered *p* value < 0.05 as statistically significant.

## Results

### Demographic and clinical characteristics of the population

**Fig. 1:**
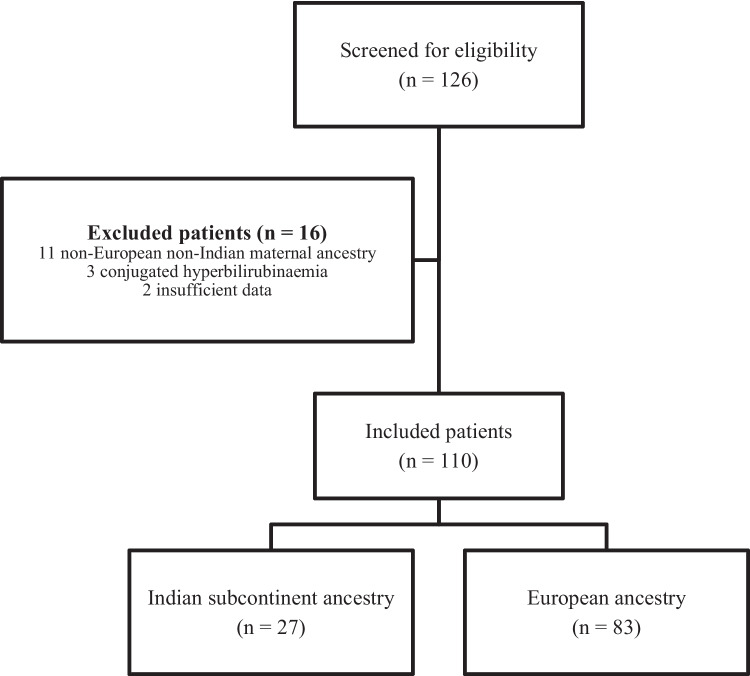
Flowchart of inclusion and exclusion process

From January 2016 to December 2021, a total of 126 newborns were admitted due to unconjugated hyperbilirubinaemia (Fig. 1). Of these, 16 were excluded due to non-European and non-Indian subcontinent ancestry, conjugated hyperbilirubinaemia or insufficient data. Of the remaining 110 patients included, 27 were in the study group (24.5%) and 83 in the control group (75.5%).

Demographic and clinical variables of the total sample and both groups are detailed in Tables 1 and 2. Apart from patients’ age and gestational diabetes history, the remainder of the collected characteristics were not found to be statistically different between groups. The age at admission was found to be higher in the study group (8 days, IQR 6–14) than in the control group (6 days, IQR 5–8) (*p* = 0.004). It was also found that the study group had a higher occurrence of gestational diabetes, with 22.2% (6/27) of the cases compared to 7.2% (6/83) in the control group (*p* = 0.03).

**Table 1:** Demographic and clinical characteristics of the population*

Characteristic	All	Indian ancestry	European ancestry	*P* value
Male, *n* (%)	69 (62.7)	17 (63.0)	52 (62.7)	0.977
Age (days)	6.0 [5.0–10.0]	8.0 [6.0–14.0]	6.0 [5.0–8.0]	0.004
Gestational age (w)	38 [37–39]	38 [38–39]	38 [37–39]	0.467
Birth weight (g)	3042.5 [2850.0–3330.0]	3040.0 [2880.0–3190.0]	3045.0 [2850.0–3370.0]	0.408
5′ APGAR score	10 [10–10]	10 [10–10]	10 [10–10]	0.902
Admission duration (days)	1.5 [1.0–3.0]	2.0 [1.0–4.0]	1.0 [1.0–2.0]	0.004
Jaundice first 24 h after birth, *n* (%)	1 (0.9)	0 (0.0)	1 (1.2)	0.999
Haemolysis, *n* (%)	2 (1.8)	1 (3.7)	1 (1.2)	0.432
Family history of neonatal hyperbilirubinaemia, *n* (%)	5 (4.5)	3 (11.1)	2 (2.4)	0.094
Family history of phototherapy, *n* (%)	4 (3.7)	2 (7.7)	2 (2.4)	0.094
Family history of haemolytic disease, *n* (%)	1 (0.9)	0 (0.0)	1 (1.2)	0.567
Scalp haematoma, *n* (%)	3 (2.7)	0 (0.0)	3 (3.6)	0.574
Feeding regimen				0.597
Exclusive breastfeeding (%)	86 (78.2)	22 (81.5)	64 (77.1)	
Exclusive formula milk (%)	3 (2.7)	0 (0.0)	3 (3.6)	
Mixed regimen (%)	21 (19.1)	5 (18.5)	16 (19.3)	

^*^Continuous variables are summarized as median values and interquartile range. Percentages may not total 100% due to rounding. The study group is represented as “Indian Ancestry” and the control group as “European Ancestry”

*5′* 5 min, *g* grams, *SGA* small for gestational age, *w* weeks

**Table 2:** Maternal demographic and clinical characteristics

Characteristic	All	Indian ancestry	European ancestry	*P* value
Maternal age (years)	31 [28–34]	30 [26–33]	32 [28–36]	0.103
Delivery				0.964
Eutocic, *n* (%)	43 (39.1)	10 (37.0)	33 (39.8)	
Instrumented, *n* (%)	44 (40.0)	11 (40.7)	33 (39.8)	
C-section, *n* (%)	23 (20.9)	6 (22.2)	17 (20.5)	
Gestational diabetes, *n* (%)	12 (10.9)	6 (22.2)	6 (7.2)	0.030
Tabagism, *n* (%)	2 (1.8)	0 (0.0)	2 (2.4)	0.999
Siblings, *n*				0.972
0, *n* (%)	67 (61.5)	16 (59.3)	51 (62.2)	
1, *n* (%)	30 (27.5)	8 (29.6)	22 (26.8)	
2, *n* (%)	11 (10.1)	3 (11.1)	8 (9.8)	
≥ 3, *n* (%)	1 (0.9)	0 (0.0)	1 (1.2)	

### Primary and secondary outcomes

**Fig. 2:**
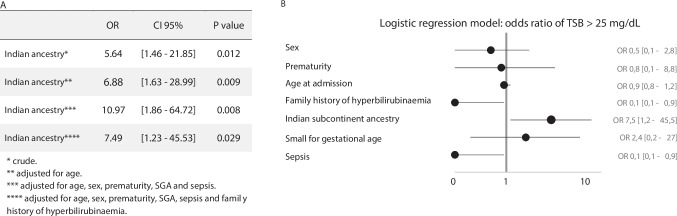
**Legend: A**, **B** Logistic regression models for hyperbilirubinaemia > 25 mg/dL, according to maternal descent. Figure 2 Logistic regression model for adjustment for confounding factors. In Fig. 2A, it is represented crude and adjusted OR for TSB > 25 mg/dL according to Indian subcontinent ancestry. In Fig. 2B, there is a graphic representation of each variable included in the final regression model, with respective OR and Confidence Intervals 95%. CI 95%, confidence interval 95%; OR, odds ratio; SGA, small for gestational age

Severe hyperbilirubinaemia occurred in 20 of 27 (74.1%) patients in the study group and in 62 of 83 (74.7%) patients in the control group (*p* = 0.948) (Table 3). Considering each component of the composite outcome, there were no significant differences in exposure to phototherapy for more than 6 h and ET therapy, but the occurrence of TSB > 25 mg/dL was significantly higher in the study group (6/27, 22.2%) comparing to the control group (4/83, 4.8%) (*p* = 0.006). After adjustment for confounding factors, Indian subcontinent ancestry predicted the occurrence of TSB > 25 mg/dL (OR 7.49, CI 95% [1.23–45.50], *p* = 0.029) (Fig. 2). Potential confounding factors considered were age at admission, sex, small for gestational age, prematurity, sepsis and family history of neonatal jaundice. Gestational diabetes was excluded from the model due to the high risk of relation with TSB > 25 mg/dL, contributing to model multicollinearity. All cases with gestational diabetes in both groups had TSB < 25 mg/dL.

**Table 3:** Primary and secondary outcomes

Outcome	All	Indian ancestry	European ancestry	*P* value
Primary outcome				
Severe hyperbilirubinaemia, *n* (%)	82 (74.5)	20 (74.1)	62 (74.7)	0.948
TSB > 25 mg/dL, *n* (%)	10 (9.1)	6 (22.2)	4 (4.8)	0.006
Phototherapy > 6 h, *n* (%)	82 (74.5)	20 (74.1)	62 (74.7)	0.948
Exchange transfusion, *n* (%)	0 (0.0)	0 (0.0)	0 (0.0)	NA
Phototherapy, *n* (%)	104 (94.5)	26 (96.3)	78 (94.0)	0.645
Phototherapy duration (hours)	8.5 [6.0–14.5]	11.0 [6.0–18.0]	8.0 [6.0–14.0]	0.252
Secondary outcome				
Admission values				
TSB (mg/dL)	20.0 [18.1–22.3]	22.0 [18.7–24.0]	19.6 [18.0–21.4]	0.013
Direct bilirubin (mg/dL)	0.6 [0.5–0.8]	0.8 [0.6–0.8]	0.6 [0.5–0.7]	0.001
Direct bilirubin/total bilirubin (%)	0.03 [0.02–0.03]	0.03 [0.03–0.04]	0.02 [0.02–0.03]	0.442
Discharge values				
TSB (mg/dL)	11.8 [10.4–13.5]	13.6 [10.9–14.6]	11.4 [10.0–12.6]	0.005
Direct bilirubin (mg/dL)	0.5 [0.5–0.6]	0.5 [0.5–0.7]	0.5 [0.5–0.6]	0.496
Direct bilirubin/total bilirubin (%)	0.04 [0.04–0.05]	0.04 [0.04–0.05]	0.05 [0.04–0.05]	0.758

*NA* not applicable, *TSB* total serum bilirubin

In respect to secondary outcomes, the study group revealed significantly higher absolute values of TSB both at admission (22.0 mg/dL, IQR [18.7–24.0] vs. 19.6 mg/dL, IQR [18.0–21.4], p = 0.013) and discharge (13.6 mg/dL, IQR [10.9–14.6] vs. 11.4 mg/dL, IQR [10.0–12.6], p = 0.005). No significant differences were observed between groups irrespective of phototherapy exposure or absolute duration.

### Genetic study

In our sample, 10 patients (9.1%) had a genetic study performed regarding the UGT1A1 gene (promotor and coding region), with 4 of them revealing alterations (Table 4). All those patients were in the study group, and the mutations identified were TA6/7 heterozygotic allele (1 patient) and UGT1A1*6 variant (c.211 G > A) homozygotic variation (3 patients). These samples had enzymatic activity tested, with 1 patient (patient 2, UGT1A1*6 variant) showing decreased UGT activity.

**Table 4:** Genetic variants reported and respective clinical profiles

Patient	Group	Genetic variants	UGT activity	TSB (mg/dL)	Age (days)	Other risk factors
Patient 1	Indian	TA7 allele, heterozygotic	Unaltered	18.45	14	No risk factors
Patient 2	Indian	c.211G > A (p.Gly71Arg), homozygotic	Decreased	30.54	9	Sepsis, family history of neonatal hyperbilirubinaemia and phototherapy
Patient 3	Indian	c.211G > A (p.Gly71Arg), homozygotic	Unaltered	20.95	17	Gestational diabetes
Patient 4	Indian	c.211G > A (p.Gly71Arg), homozygotic	Unaltered	23.72	18	No risk factors

*UGT* 5′-diphosphate-glucuronosyltransferase, *TSB* total serum bilirubin

## Discussion

In the present study, Indian subcontinent maternal ancestry was associated with a significantly higher risk of developing neonatal hyperbilirubinaemia with TSB > 25 mg/dL compared to European ancestry, maintained after adjustment for confounding factors (OR 6.97, CI 95% [1.15–42.24], *p* = 0.026). Our data suggests that these infants have an increased risk for severe hyperbilirubinaemia. To our knowledge, this is the first study regarding hyperbilirubinaemia severity comparing neonates from European and Indian ancestry. Although clinical practice guidelines have been evolving towards a less interventive approach in neonatal hyperbilirubinaemia, levels of TSB > 25 mg/dL (427 mmol/L) are close to the ET threshold in term-newborns older than 72 h [10]. Whether a less interventive approach applies adequately to this subset of patients is still to be answered.

As mentioned earlier, severe hyperbilirubinaemia may contribute to long-term neurodevelopment impairment, with or without acute bilirubin encephalopathy [2]. In our sample, newborns from Indian subcontinent ancestry were older at admission, had longer hospital stays and had higher levels of TSB (both at admission and discharge). We conjecture that those findings might have played a role in hyperbilirubinaemia severity. A study from Boo et al. also reports later diagnosis and age at admission as a risk factor for severe hyperbilirubinaemia [20]. Moreover, our study does not report transcutaneous bilirubin (TcB) measurement. In fact, TcB is a non-invasive technique that helps to accurately estimate TSB levels, particularly when TSB < 15 mg/dL [21–23]. Different ethnicities correlate with different TcB level normograms, making it a useful technique in clinical practice for a multitude of backgrounds [24]. Nonetheless, current evidence and guidelines recommend that every TcB > 15 mg/dL or near the treatment threshold should be confirmed with TSB measurement [10]. As our cohort includes only patients requiring treatment, solely TSB levels were reported. Future technological advances might allow TcB to be a reliable tool for higher TSB levels, permitting a less invasive approach.

Prior to the study, we conjectured that higher levels of bilirubin in one group would result in longer periods of phototherapy and higher rates of ET. Surprisingly, no differences were verified considering phototherapy duration and ET performance (Table 3). Concerning phototherapy, we found multiple electronic records to be incomplete. On the other hand, 6 of our patients reached the ET threshold (like patient 4 from Table 4), but none had an escalation of care. ET is a complex, expensive and resource-consuming technique, recommended by the AAP in cases of extreme hyperbilirubinaemia [10]. We think that factors like the need for a neonatal intensive care unit, inherent risks (like infection and haemorrhage) and high costs of ET might interfere with escalation of care. In fact, one study reported the non-inferiority of intensive phototherapy compared to ET in the treatment of extreme hyperbilirubinaemia [12]. Although it had a 3-year period follow-up after treatment, it was a retrospective cohort and not a randomized controlled trial, enlightening many limitations that should not be ignored. To this date, AAP recommends ET as first-line therapy in extreme hyperbilirubinaemia, and we found insufficient data supporting intensive phototherapy preference over ET in those cases [10].

Regarding the cause, many studies point out genetic polymorphisms as a base for the higher incidence of hyperbilirubinaemia in Asian-descendent communities, particularly in countries like China, Japan and the Philippines [11, 20]. Most studies highlight genetic polymorphisms as the cause to hyperbilirubinaemia, mostly with mutations involving UGT1A1 and SLCO1B1 [1]. In our study, only a small subset of patients underwent genetic testing, four of them revealing genetic mutations (Table 4). One had promoter gene mutation (TA7/TA6 heterozygotic allele mutation) of uncertain clinical significance, and three others had c.211 G > A mutations (known for causing jaundice). Boo et al. mentioned previous reports mentioning a correlation between severe hyperbilirubinaemia and variations in UGT1A1, SLCO1B1 and glucose-6-phosphate dehydrogenase genes [20]. One case–control study from Yang et al. identified a higher rate of c.211 G > A in the UGT1A1 gene in neonates with severe hyperbilirubinaemia compared to controls without hyperbilirubinaemia, supporting our findings [15]. We acknowledge that genetic polymorphisms regarding bilirubin conjugation might play a significant role in hyperbilirubinaemia development. However, in our sample, only one patient had a mutation with identified lower enzymatic activity (Table 4). Aside from better genetic characterization, other factors like maternal dietary habits, delayed healthcare admission or underrecognized clinical jaundice should be addressed in the future as they might influence severe hyperbilirubinaemia. Gestational diabetes was more frequent in the study group than in the control group, although none presented TSB > 25 mg/dL. A higher incidence of both type 2 diabetes mellitus and gestational diabetes in the Indian population has already been reported [25]. As gestational diabetes is a known risk factor for neonatal hyperbilirubinaemia, in our sample, it did not translate to increased clinical severity. Further research should address gestational diabetes’ role in hyperbilirubinaemia severity.

Long-term follow-up data was unfortunately unavailable for most patients. We believe that prospective long-term follow-up data should help to clarify if newborns from Indian subcontinent ancestry are more susceptible to long-term neurodevelopmental compromise compared to European ancestry newborns. Consequently, we propose two opposite research pathways in the future. If the study group reveals higher neurodevelopmental compromise, the investigation should address more precocious treatment, careful pre and after-birth vigilance and neurodevelopmental intervention. However, if these higher TSB levels do not reflect in neurodevelopmental co-morbidity, overtreatment and over-admission should be avoided, guiding research towards population-based adjusted thresholds for phototherapy and ET in this subset of patients.

## Limitations

There are several limitations to report in the present study. First, the retrospective design led to incomplete information in health records. Although most data was available, certain records were missing details, including past personal and family history and phototherapy duration/modality. Furthermore, paternal nationality was not available in most records, making it impossible to include in the analysis and undermining both parent vs. one-parent ancestry impact on hyperbilirubinaemia. We believe that the implementation of standardized daily registration including these data could help overcome those limitations. Secondly, the absence of sample size calculation might lead to a small sample size and interfere with data and conclusions’ generalizability. Multicentric studies could allow more patients to be enrolled and produce more robust evidence. Finally, the absence of standardized follow-up data compromised the long-term evaluation of neurological complications, which could be obtained by prospective studies.

## Conclusions

In our retrospective cohort of patients, newborns from Indian subcontinent mothers were at higher risk of developing hyperbilirubinaemia > 25 mg/dL than European ancestry newborns (adjusted OR 7.49, CI 95% [1.23–45.50]). Severe hyperbilirubinaemia might lead to long-term neurological consequences, which, if confirmed in this subset of patients in prospective studies, should lead to better pre and post-natal vigilance. If no long-term consequences are verified, more suitable phototherapy and ET thresholds should take place for this subset of patients, avoiding overtreatment and over-admission.

## Data Availability

The data that support the findings of this study are available from the corresponding author upon reasonable request.

## Abbreviations

AAP: American Academy of Pediatrics
BIND: Bilirubin-induced neurologic dysfunction
CI: Confidence interval
ET: Exchange transfusion
IQR: Interquartile range
OR: Odds ratio
SD: Standard deviation
STROBE: Strengthening the Reporting of Observational Studies in Epidemiology
TSB: Total serum bilirubin
UGT: 5′-Diphosphate-glucuronosyltransferase
